# Survey of single-nucleotide polymorphisms in the gene encoding human deoxyribonuclease I-like 2 producing loss of function potentially implicated in the pathogenesis of parakeratosis

**DOI:** 10.1371/journal.pone.0175083

**Published:** 2017-04-10

**Authors:** Misuzu Ueki, Haruo Takeshita, Natsuko Utsunomiya, Takanao Chino, Noritaka Oyama, Minoru Hasegawa, Kaori Kimura-Kataoka, Junko Fujihara, Reiko Iida, Toshihiro Yasuda

**Affiliations:** 1 Department of Medical Genetics and Biochemistry, Faculty of Medical Sciences, University of Fukui, Eiheiji, Fukui, Japan; 2 Department of Legal Medicine, Shimane University School of Medicine, Enya, Izumo, Japan; 3 Department of Dermatology, Faculty of Medical Sciences, University of Fukui, Eiheiji, Fukui, Japan; 4 Department of Life Sciences, Faculty of Medical Sciences, University of Fukui, Eiheiji, Fukui, Japan; University of Queensland Diamantina Institute, AUSTRALIA

## Abstract

Dysfunction of DNase I-like 2 (DNase 1L2) has been assumed to play a role in the etiology of parakeratosis through incomplete degradation of DNA in the epidermis. However, the pathogenetic background factor for such pathophysiologic conditions remains unknown. In this context, non-synonymous single-nucleotide polymorphisms (SNPs) in *DNASE1L2* that would potentially result in loss of *in vivo* DNase 1L2 activity might serve as a genetic risk factor for such pathophysiologic conditions. Our aim was to effectively survey the non-synonymous SNPs of *DNASE1L2* that would produce a loss-of-function variant of the enzyme together with a genetic distribution in the various populations. Here, the effects of all of the SNPs predicted by PolyPhen-2 analysis to be “probably damaging” (score = 1.000), and derived from frameshift/nonsense mutations, on the activity of DNase 1L2 were examined using the corresponding DNase 1L2 variants expressed in COS-7 cells. Genotyping of these SNPs was also performed in three ethnic groups including 14 different populations. Among the 28 SNPs examined, the minor allele of 23 SNPs was defined as a loss-of-function variant resulting in loss of DNase 1L2 function, indicating that Polyphen-2 analysis could be effective for surveys of at least non-synonymous SNPs resulting in loss of function. On the other hand, these minor alleles were not distributed worldwide, thereby avoiding any marked reduction of the enzyme activity in human populations. Furthermore, all of the 19 SNPs originating from frameshift/ nonsense mutations found in *DNASE1L2* resulted in loss of function of the enzyme. Thus, the present findings suggest that each of the minor alleles for these SNPs may serve as one of genetic risk factors for parakeratotic skin diseases such as psoriasis, even though they lack a worldwide genetic distribution.

## Introduction

It has been shown that degradation of DNA is an indispensable part of the terminal differentiation program of keratinocytes, thus contributing to keratinocyte cornification, which helps to maintain the integrity of the epidermis [1]. It has been postulated that deoxyribonuclease I-like 2 (DNase 1L2) and three prime repair exonuclease 2 (TREX2) in the epidermis, and DNase II (EC3.1.21.1) on the skin surface, are the nucleases likely responsible for such DNA degradation [2–5]. DNase 1L2 has been identified as a member of the DNase I family [6–8]. Unlike the high activity shown by the other members at neutral pH, DNase 1L2 uniquely exhibits optimal activity under acidic conditions, and possesses a proline-rich extra sequence located in the central region of the protein; A splicing variant of the DNase 1L2 transcript in which this proline-rich extra sequence is deleted has been found in peripheral blood leukocytes [9]. It is especially noteworthy that, in contrast to the other member enzymes [8], DNase 1L2 is considerably more abundant in the skin than in any other organ at both the mRNA and protein levels, and its expression correlates with the terminal differentiation of epidermal keratinocytes [10]. For this reason, DNase 1L2 has been assumed to serve as a keratinocyte-specific endonuclease possibly playing an essential role in DNA degradation. Fischer et al. [10] have demonstrated that expression of the DNase 1L2 gene is obviously reduced in epidermis affected by parakeratosis, as is the case in psoriasis lesions, which are characterized by aberrant retention of nuclear chromatin, including DNA, known as parakeratosis. Furthermore, suppression of DNase 1L2 by RNAi in a human skin model causes parakeratocytosis. These findings strongly indicate that dysfunction of DNase 1L2, specifically in the epidermis, may play a role in the etiology of parakeratosis through incomplete degradation of DNA. Furthermore, since knockout of the DNase 1L2 gene in mice leads to retention of high amounts of nuclear DNA in hair and nails, DNase 1L2 might contribute specifically to nuclear DNA degradation in differentiation-associated cell death in various keratinocyte lineages [11]. Recently, it has been demonstrated that DNase 1L2, together with DNase I, are indispensable for nuclear DNA in sebocytes [12].

As these findings seem to suggest that substantial reduction or abolishment of DNase 1L2 activity *in vivo* might be related to the pathogenesis of skin diseases through incomplete degradation of nuclear DNA, we have focused upon non-synonymous single-nucleotide polymorphisms (SNPs) in the human DNase 1L2 gene (*DNASE1L2*) that affect the *in vivo* level of DNase 1L2 activity through the corresponding amino acid substitution, as a pathogenetic background factor [13–16]. Although a large number of non-synonymous SNPs in *DNASE1L2* that would likely affect the catalytic activity have been registered in the NCBI dbSNP database (http://www.ncbi.nlm.nih.gov/projects/SNP), it remains to be clarified whether the functionality of DNase 1L2 would be damaged by many of these SNPs.

Therefore, our aim in the present study was to effectively survey the non-synonymous SNPs of *DNASE1L2* that would reduce the activity of the enzyme, and thus act as potentially pathogenetic SNPs conferring a genetic predisposition to parakeratosis. For this purpose, Polymorphism Phenotyping-2 (PolyPhen-2) [17], a software program useful for predicting the effects of SNPs on protein function, was used to select candidate SNPs leading to loss of function of DNase 1L2. Subsequently, for 28 non-synonymous *DNASE1L2* SNPs that were considered likely to impair the enzyme activity, the effects of the corresponding amino acid substitution on the catalytic activity and the genetic distribution of the SNPs in 14 different populations worldwide derived from 3 ethnic groups were examined, thereby revealing the functional and genetic aspects of each SNP. Furthermore, we examined all of the SNPs originating from frameshift/nonsense mutations in the gene to determine whether they could lead to loss of enzyme function. As a result, the minor alleles of the 23 non-synonymous SNPs and the 19 SNPs originating from frameshift/ nonsense mutations found in *DNASE1L2* were found to result in loss of function of the enzyme. Our findings would help to clarify their role in genetic disposition to parakeratocytosis.

## Materials and methods

### Prediction of the functional effect of non-synonymous SNPs in *DNASE1L2* using PolyPhen-2

In order to predict possible changes in the function of DNase 1L2 due to amino acid substitutions resulting from the non-synonymous SNPs, Polyphen-2 (http://genetics.bwh.harvard.edu/pph2/index.shtml) was used. Since, in the database, all of the non-synonymous SNPs identified in *DNASE1L2* have exhibited low heterozygosity, the HumDiv-trained model was used for prediction [18]. The PolyPhen-2 score corresponds to the probability of a substitution being damaging, and ranges from 0.0 (tolerated) to 1.0 (deleterious); the prediction outcome can be presented as “benign”, “possibly damaging” or “probably damaging”.

### Construction of expression vectors encoding the amino acid-substituted DNase 1L2 derived from a minor allele in each SNP

The recommendations for description of sequence variants (http://www.hgvs.org) was used for each SNP nomenclature, in which the sequence of DNase 1L2 (NCBI Reference Sequence: NM_001301680.1) has been used as the coding DNA Reference Sequence. Nucleotide and amino acid residues were numbered from the 5'-terminus of the translation initiation codon and the N-terminal amino acid residue of the precursor protein, respectively.

A DNA fragment containing the entire coding sequence of human DNase 1L2 cDNA was prepared by reverse transcription (RT)-PCR amplification of the total RNA of human peripheral leukocytes (Clontech, Palo Alta, CA, USA) using an Expanded High Fidelity PCR System (Roche Diagnosis, Mannheim, Germany); a set of two primers, 5'-**GGATCC**CACCCACATCCAAGGCGGCAGCG-3' (sense) and 5'-**CTCGAG**TCATCGGTGGAACTTGAG-3' (antisense), corresponding to the sequences of positions -54–-31and 883–907 in the DNase 1L2 cDNA, in which the restriction site of *Eco*RI and *Xho*I as shown in bold were introduced, respectively, were used. After digested with *Eco*RI and *Xho*I, the amplified DNA fragment was ligated into pcDNA3.1(+) (Invitrogen, San Diego, CA, USA). Since the inserted DNA fragment was confirmed by sequencing of the construct to be derived from the predominant haplotype for the SNPs, the construct was used as a wild type DNase 1L2 expression vector. In order to construct expression vectors encoding the amino acid substituted DNase 1L2 derived from a minor allele in each SNP, the site-directed mutagenesis using KOD-Plus Mutagenesis Kit (Toyobo Co. Ltd., Osaka, Japan) with the wild type construct as a template was separately employed; e.g., the R23P construct of DNase 1L2, in which the Arg residue at position 23 in the protein is replaced by Pro derived from the minor allele, corresponds to SNP p.Arg23Pro (rs767698904). Furthermore, 10 variant constructs for frameshift/nonsense mutations in the gene, and those for the short and the related deleted form of DNase 1L2 [9] were separately prepared in the same manner as above. Nucleotide sequences of all the constructs were confirmed by DNA sequence analysis. Two different clones derived from each construct used for transfection were purified using the Plasmid Midi kit (Qiagen).

A RT-PCR analysis of transcripts from *DNASE1L2* in human skin was performed according to the previous method [9] using the total RNA of human normal skin (BioChain Institute, Newark, CA).

### Transient expression of the expression vectors and assay for DNase 1L2 activity

COS-7 cells maintained in Dulbecco’s modified Eagle medium containing 1 mM L-glutamine, 50 U/ml penicillin, 50 μg/ml and 10% (v/v) fetal calf serum at 37°C under 5% CO_2_ in air were transiently transfected with 2 μg of each expression vector using Lipofectamine 2000 reagent (Invitrogen) according to the method described previously [19]. At 48 hours later, the cells and the conditioned medium were recovered. The DNase 1L2 activity in the conditioned medium was assayed by the single radial enzyme diffusion (SRED) method using a LAS-3000 imaging analyzer (Fuji Film, Tokyo, Japan) according to our previous report [16]. The mean activity of the amino acid—substituted form derived from 4 transfections using 2 different clones derived from each construct was expressed relative to that of the wild type; the relative activity is expressed as mean ± standard deviation.

The activity of each amino acid-substituted DNase 1L2 was compared with that of the wild-type by means of the unpaired, Student’s *t*-test.

### DNA samples

Blood or bloodstain samples randomly collected from healthy subjects (n = 1,752) derived from 14 different populations including 3 ethnic groups were used for extraction of genomic DNA by means of a QIAamp DNA Mini Kit (Qiagen, Chatsworth, CA, USA). The Asian populations included 112 Mongolians (Ulaanbaatar, Mongol), 110 Japanese (Shimane prefecture), 352 Koreans (Busan, South Korea), 193 South Chinese (Shenyang and Guangzhou of China), 153 Tibetans (Katmandu of Nepal), 40 Tamangs (Kotyang of Nepal) and 83 Sri Lanka (35 Tamils and 48 Sinhalese, Kandy of Sri Lanka); the Caucasian population included 68 Germans (Munich), 136 Turks (Adana area, Southern Turkey) and 199 Mexicans (60 Mestizo, 88 Nahuas and 51 Huicholes); the African populations included 105 Ghanaians (Ghana), 126 Ovambos (Bantusin, Namibia) and 75 Xhosas (Cape Town, South Africa). All of these populations were uniform in terms of ethnic variation. Written informed consent was obtained from each subject, and this study was approved by the Human Ethical Committees of the institute (the approval number 1024 for the Human Genome and Genetics Analysis Study).

### Genotyping of each SNP in the DNase 1L2 gene

In the present study, PCR-restriction fragment length polymorphism (RFLP) method [20] was used in order to determine the genotype of each SNP in *DNASE1L*2. The corresponding PCR primers for the genotyping was designed on the basis of the nucleotide sequence of the human DNase 1L2 gene (GenBank: accession number no. AY298958.1); the sequence of the primers, annealing temperature and restriction enzyme used for the analysis of each SNP are shown in S1 Table. When the substitution site in the SNPs neither suppressed nor created any known restriction enzyme recognition sites, a mismatched PCR-RFLP technique [20] was employed for this genotyping; incorporation of a deliberate mismatch nucleotide(s) close to the 3'-terminus of the PCR primer allowed to create a novel restriction enzyme recognition site in the amplified DNA fragment.

PCR amplification was performed as described in detail previously [16,19]. The appearance of the expected product, as shown in S1 Table, derived from the respective alleles in each SNP, allowed us to determine the genotypes easily; in SNP p.Arg23Pro, the 128-bp amplified product derived from the *G* allele was completely digested with *HinfI* to yield a103-bp fragment, whereas that from the *C* allele did not yield such fragment.

The validity of the genotyping results obtained by these methods was confirmed according to the previous procedure [16].

## Results and discussion

### Evaluation of the functional effect of non-synonymous SNPs in *DNASE1L2* using PolyPhen-2

Previously, we have demonstrated the effects of 43 non-synonymous SNPs in *DNASE1L2* in terms of the level of DNase 1L2 activity [13–16]. In the present study, the functional effects of amino acid substitutions resulting from these non-synonymous SNPs were predicted using Polyphen-2 (Table 1). Based on the Polyphen-2 scores, all of the activity-abolishing SNPs were predicted to be “probably damaging”, whereas no activity-abolishing SNPs were found among all those predicted to be “benign” and “possibly damaging”. Furthermore, among the amino acid substitutions resulting from the SNPs assumed to be “probably damaging” (score <1.000), several that did not alter the DNase 1L2 activity were found. Thus, SNPs abolishing the activity were successfully predicted to be “probably damaging” (score = 1.000). These findings permitted us to postulate that at least the SNPs causing loss of function of DNase 1L2, and serving as genetic risk factors for the pathogenesis of parakeratosis, could be estimated from Polyphen-2 scores corresponding to “probably damaging” (score = 1.000) with a high predictive accuracy.

**Table 1:** Evaluation on a prediction^a^ of functional effect of non-synonymous SNPs^b^ in *DNASE1L2* by PolyPhen-2.

Prediction outcome	Total	No Effect	Elevating the activity	Reducing the activity	Abolishing the activity
Benign	21	11	2	8	0
Possibly damaing	6	1	1	4	0
Probably damaging^c^ (score<1.000)	6	4	0	2	0
Probaly damaging^c^ (score = 1.000)	10	0	0	3	7

^a^The HumDiv-trained model was used for evaluating rare allele in each non-synonymous SNPs.

^b^Forty-three non-synonymous SNPs have been examined on effect through the corresponding amino acid substitution on the level of the DNase 1L2 activity in our previous studies [13–16].

^c^The non-synonymous SNPs predicted as “probably damaging” are classified into two categories based on the score (1.000 or <1.000) corresponding to the probability of the substitution being damaging.

### Effect of the amino acid substitution derived from each of the non-synonymous SNPs classified as “probably damaging” (score = 1.000) on DNase 1L2 activity

In order to survey functional SNPs in *DNASE1L2* as efficiently as possible, and to re-evaluate the damaging effect of the SNPs predicted by PolyPhen-2, all of the 28 SNPs assumed to be “probably damaging” among the non-synonymous SNPs registered in the NCBI dbSNP, based on a score of 1.000, except for 10 SNPs examined previously [16], were selected in the present study.

**Fig 1:**
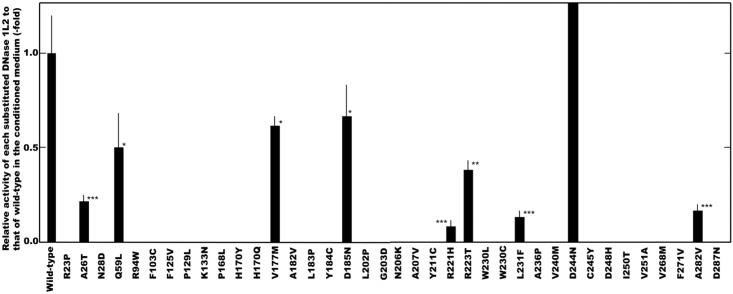
Effect of the amino acid substitution derived from each non-synonymous SNPs examined on the DNase 1L2 activity.

**Fig 2:**
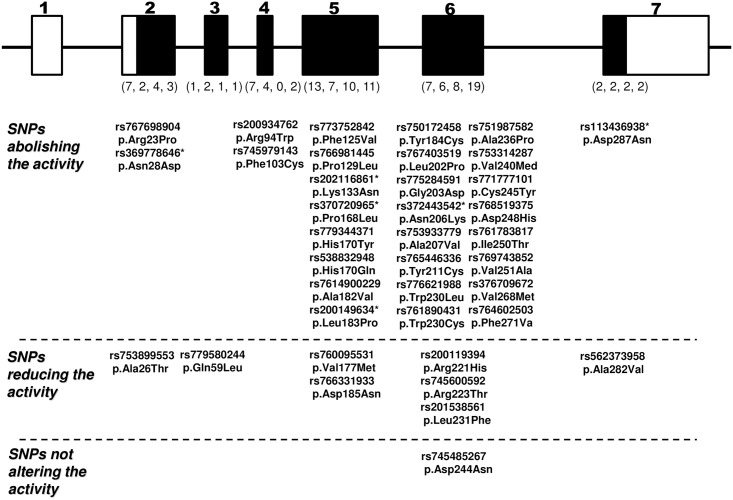
All of the 38 non-synonymous SNPs predicted to be “probably damaging (score = 1.000) in the human DNase 1L2 gene.

First, we examined the effect of the amino acid substitution corresponding to each of these non-synonymous SNPs in terms of DNase 1L2 activity. As shown in Fig 1A and 1B, compared with the activity level of the wild-type DNase 1L2, the 38 “probably damaging” SNPs, including 10 SNPs examined previously [16], in the DNase 1L2 gene were classified into only one SNP not affecting the activity level, and 8 that reduced it, whereas the remaining 29 SNPs abolished the activity completely (Fig 2). It was noteworthy that, since each activity originating from the amino acid-substituted DNase 1L2 corresponding to the latter 29 SNPs could be not detected under our assay conditions, the minor allele in each SNP was identified as a loss-of-function variant completely abolishing DNase 1L2 activity. Furthermore, among the 8 SNPs that reduced the activity, the amino acid substitution corresponding to SNPs p.Ala26Thr, p.Arg221His, p.Leu231Phe and p.Ala282Val markedly reduced the activity to less than about 20% of that of the wild-type. The DNase 1L2 activity derived from only D244N was similar to that of the wild type, indicating that irrespective of classification as a “probably damaging” SNP, substitution of the Asp residue by Asn at position 244 had little effect on the activity. The fact that the Asp residue is not entirely conserved in the human DNase I family, His or Asn being alternatively situated at the corresponding position [7], could account for this lack of effect of the SNP on the activity.

The DNase 1L2 activities in the conditioned medium from the cells transfected with each construct were determined by SRED method [16]. Relative activities of the amino acid substituted DNase 1L2 derived from a minor allele of each SNP to that of the wild-type enzyme were presented. Levels of the DNase 1L2 activity derived from the SNPs marked with asterisk were significantly reduced; P-values calculated for the differences between the activities of the substituted and wild-type DNase 1L2 using unpaired Student’s *t* test: *p<0.05, **p<0.005, ***p<0.001. On the other hand, substitution of Asp by Asn in DNase 1L2, corresponding to D244N, did not increase the activity level significantly (1.3±0.36). Bars show standard deviation (n = 4); no bar exhibit no activity detectable under our assay conditions.

The structural organization of the human DNase 1L2 gene shown here is based on GenBank: accession number no. AY298958.1. Exons are shown by solid boxes, in which solid and clear boxes correspond to the translated and untranslated regions of the mRNA, respectively. The locations of each of the SNPs in the exons and ID number in the NCBI database are shown. According to alterations in the levels of DNase 1L2 activity resulting from the corresponding amino acid substitution, the 38 non-synonymous SNPs in the gene were classifiable into 1 SNPs not affecting the enzyme activity, 8 reducing the activity, and 29 abolishing the activity, the last ones of which might be served as a pathogenic SNP producing loss of function of DNase 1L2. Numbers of the SNPs predicted to be “benign”, “possibly damaging”, “probably damaging (score<1.000)” and “probably damaging (score = 1.000), respectively, are shown in parenthesis under each exon. Inactivating SNPs marked with asterisk were identified in our previous study [16].

Polyphen-2, available as software and via a Web server, is a useful tool for predicting the possible impact of an amino acid substitution on the function of a human protein, being indispensable for interpretation of genetic variants resulting from non-synonymous SNPs, and especially for identification of pathogenetic SNPs [17, 18]. Our present analysis of the DNase 1L2 protein itself allowed us to substantiate the accuracy of the predicted functional impact of an amino acid substitution corresponding to any given non-synonymous SNP using Polyphen-2. Previously, we demonstrated that at least the SNPs resulting in loss of function of human DNase family genes encoding DNases I, 1L3 and II can be estimated by Polyphen-2 scores corresponding to “probably damaging” with high predictive accuracy [21]. Thus, Polyphen-2 analysis to predict the possible functional impact of SNPs is sufficiently reliable for surveying at least non-synonymous SNPs resulting in loss of function.

### Genetic heterogeneity of non-synonymous SNPs abolishing the activity in 14 different human populations

We next examined the genetic distribution of all of the 23 non-synonymous SNPs in *DNASE1L2* newly identified above abolishing the activity of DNase 1L2 in the 14 different populations, including 3 ethnic groups; the distribution of the genotypes and allele frequencies for each SNP were summarized in S2 Table. As a result, all of the SNPs exhibited a monoallelic distribution in all of the populations examined; only one homozygous genotype of the predominant allele, which produces the wild-type DNase 1L2 protein, was found. The fact that the minor allele producing the loss of function variant was not observed in any of the SNPs in the 14 study populations obviously indicates that these “probably damaging” SNPs have extremely low genetic diversity. In the Exome Aggregation Consortium (ExAC) database (http://exac.broadinstitute.org), where frequency data for all the activity-abolishing SNPs we identified are available, except for SNPs p.Phe103Cys, p.Phe125Val, p.His170Gln and p.Asp187Asn, the minor allele of each SNP exhibits an extremely low frequency, being consistent with our findings. Therefore, it is plausible to assume that the minor allele responsible for loss of function of DNase 1L2 for each SNP has not been distributed across wide populations during the course of human evolution, thereby avoiding any marked reduction of the enzyme activity in human populations, as already demonstrated for other members of the human DNase family: DNase I [22], DNase 1L1 [23], DNase 1L3 [22] and DNase II [24].

### Effect of frameshift/nonsense variants of *DNASE1L2* on DNase 1L2 activity

**Fig 3:**
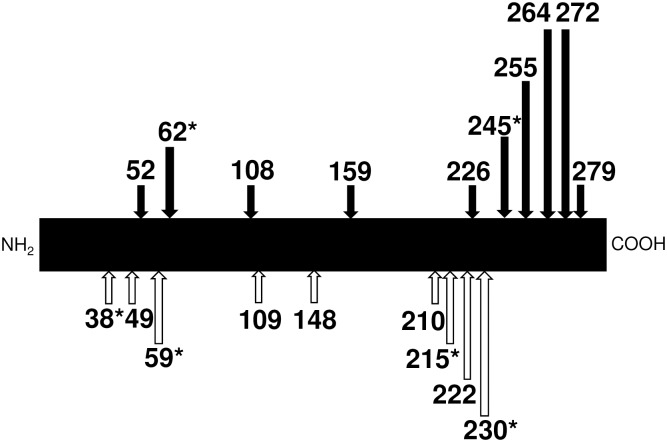
All the SNPs originated from frameshift/nonsense mutations in the DNase 1L2 gene. The position of the amino acid residue, in the codon of which mutations occur, are shown on the precursor of the DNase 1L2 protein presented as a solid bar. When the DNase 1L2 activities of the conditioned medium from the cells transfected with the constructs corresponding to each mutation marked with solid arrow were determined using the SRED method [16], no activity from all the construct examined could be detected under our assay conditions. SNPs marked with asterisk are generated by nonsense mutation.

Although 13 frameshift and 6 nonsense variants in the DNase 1L2 gene are registered in the NCBI dbSNP, their effects on DNase 1L2 activity have remained unknown (Fig 3). We prepared a series of expression constructs corresponding to 8 frameshift (p.Gly52fs, p.Asp108fs, p.Ala159fs, p.Glu226fs, p.Ala255fs, p.Gln264fs, p.Gln272fs and p.Gln279fs) and 2 nonsense (Arg62X and p.Cys245X) variants, whose corresponding amino acid positions extended over the amino acid sequence of the enzyme. These constructs were transfected into COS-7 cells, and the DNase 1L2 activity in the conditioned medium of each preparation was determined by the SRED method. It was found that none of the frameshift and nonsense constructs exhibited DNase 1L2 activity, demonstrating that all of the alleles corresponding to these frameshift/nonsense variants led to loss of function of DNase 1L2. Especially, considering that the p.Cys245X and p.Gln279fs variants of DNase 1L2 showed no detectable activity, it was suggested that a stretch of at least 19 amino acids from the C-terminus of the protein might be indispensable for the DNase 1L2 activity. Since all of the frameshift/nonsense variants examined lack this C-terminal stretch in the protein, it is reasonable that the cells transfected with the other frameshift/nonsense constructs expressed no DNase 1L2 activity. Thus, from these findings, it is plausible to conclude that all of the 19 SNPs originating from frameshift or nonsense mutations in *DNASE1L2* result in loss of function of the enzyme due to the absence of this C-terminal stretch, and that they can be considered pathogenetic SNPs. In the NCBI dbSNP database, these SNPs exhibit extremely low heterogeneity.

**Fig 4:**
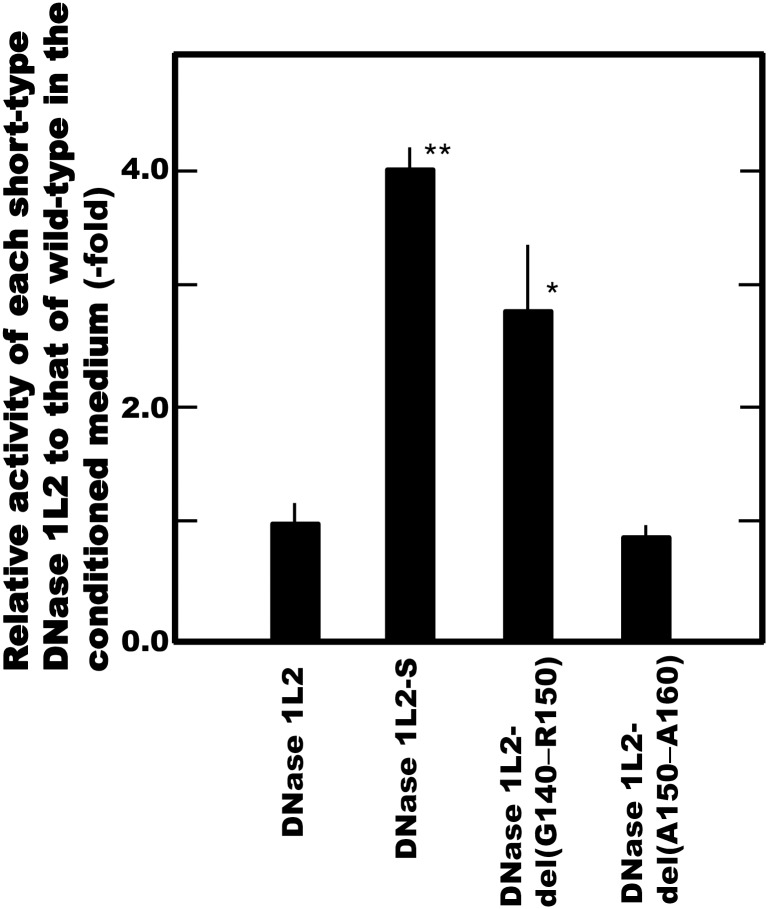
Effect of deletion of the proline-rich domain from the DNase 1L2 protein on the activity.

The short form of DNase 1L2 protein, known as DNase 1L2-S, from which the proline-rich domain (Gly140 to Ala160) of wild-type DNase 1L2 is deleted, is reportedly expressed in human peripheral blood leukocytes [9]. We prepared expression vectors corresponding to DNase 1L2-S, and two deletion forms—del(G140-R150) and del(A151-A160)–lacking the region from Gly140 to Arg150 and from Ala151 to Ala160, respectively. The DNase 1L2 activity derived from the cells transfected with these constructs was then compared with that of the wild type (Fig 4). The levels of DNase 1L2 activity derived from DNase 1L2-2 and -del(G140-R150) were found to be about 4- and 3-fold higher than that of the wild type, respectively, whereas the activity of DNase 1L2-del(A151-A160) was similar to that of the wild type. Thus, it was clarified that deletion of the proline-rich region (especially the anterior half) of the original DNase 1L2 protein increased the activity of the enzyme. These findings are consistent with the suggestion of Shiokawa et al. [9] that the presence of the proline-rich domain close to the active His residue is likely to interfere with enzyme-substrate interaction or disrupt the higher structure of the protein, thereby reducing the activity of DNase 1L2. However, since no DNA fragment amplified from the DNase 1L2-S in the RT-PCR analysis of the normal human skin was observed (data not shown), we conclude that the original DNase 1L2, and not its short form, might be the endonuclease playing an essential role in DNA degradation during the terminal differentiation of epidermal keratinocytes.

The DNase 1L2 activity derived from the transfected cells with each construct corresponding to DNase 1L2-S, and 2 deleted forms, del(G140-R150) and del(A151-A160), were compared to that of the wild type. Levels of the DNase 1L2 activity derived from DNase 1L2-S and -del(G140-R150) were significantly elevated; P-values calculated for the differences between the activities of the deleted and wild-type DNase 1L2 using unpaired Student’s *t* test: *p<0.005, **p<0.001. Bars show standard deviation (n = 4).

### Clinical implications of SNPs producing loss of function of DNase 1L2

Fisher et al. [10] demonstrated that nuclear DNA was retained in the stratum corneum of keratinocytes treated with DNase 1L2-specific RNAi, leading to efficient suppression of DNase 1L2 expression, resembling a pathological condition known as parakeratosis, found in inflammatory, hyperkeratotic diseases such as psoriasis. It is therefore plausible to consider that DNase 1L2 might be the endonuclease primarily responsible for nuclear DNA degradation during the differentiation of epidermal keratinocytes. With regard to other members of the human DNase family, we have reported that subjects heterozygous for a minor allele attributable to a non-synonymous SNP in the DNase I gene had markedly lower *in vivo* DNase activity than subjects without the corresponding allele [25]. Furthermore, some alleles of regulatory SNPs that reduce the promoter activity of the human DNase II gene result in lower *in vivo* enzyme activity [26]. These findings suggest that functional SNPs abolishing or substantially reducing the activity, or regulatory SNPs reducing the promoter activity, would likely affect DNase 1L2 in a similar way. In fact, novel nonsense [27] and missense [28] mutations in the DNase I gene abolishing and reducing the activity of DNase I, respectively, have been identified in patients with autoimmune disease. Also in patients with systemic lupus erythematosus, a null mutation in the DNase 1L3 gene resulting from a 1-bp deletion and homozygosity for a missense mutation, resulting in enzyme inactivation, have been reported [29]. In the present study, based upon the prediction of a possible functional effect of the non-synonymous SNPs in *DNASE1L2*, we were able to identify 29 pathogenetic SNPs resulting in loss of function of the enzyme (Figs 1 and 2). Also, it seems likely that all of the 19 variants resulting from frameshift or nonsense mutations will be confirmed to have a pathogenetic effect (Fig 3). Therefore, subjects who are homozygous or show compound heterozygosity for each of the minor alleles of these 48 pathogenetic SNPs, resulting in a DNase 1L2-deficient phenotype, will likely have lower levels of *in vivo* DNase 1L2 activity than subjects with other genotypes, leading to dysfunctional degradation of nuclear DNA during keratinocyte differentiation. Considering that reduction of *in vivo* DNase 1L2 activity could be associated with the etiology of epidermal parakeratosis [5], it can be postulated that the 48 pathogenetic SNPs that have been identified in *DNASE1L2* might contribute significantly to a genetic predisposition to failure of differentiation-associated cell death in various keratinocyte lineages through incomplete degradation of nuclear DNA, thereby leading to the development of parakeratotic conditions such as psoriasis.

During the formation of hair fibers from keratinocytes in the hair follicle, which involves breakdown of nuclear DNA, DNase 1L2 is thought to play an essential role in DNA removal, thus establishing the full mechanical resilience of hair [11]; A polymorphic trait for completeness of DNA degradation during cornification of the hair, being possibly useful for forensic purpose, has been demonstrated [30]. It has also been reported that DNase 1L2 might contribute to nail plate formation through differentiation-associated degradation of nuclear DNA in epidermal keratinocytes [31], and that the enzyme secreted from these keratinocytes may restrict the formation or maintenance of cutaneous bacterial biofilms through degradation of exogenous DNA on the skin surface [32]. Therefore, it can be assumed that the mechanical resilience of hair, formation of the nail plate, or the anti-biofilm defense function of the stratum corneum might be impaired by the various minor alleles of these pathogenetic SNPs in *DNASE1L2*.

In the present study, we have extensively expanded our previous studies on the functional and genetic characteristics of non-synonymous SNPs in the human DNase 1L2 gene [13–16]. All of the 35 non-synonymous SNPs examined previously, irrespective of whether or not they might affect the functionality of the enzyme, exhibited an extremely low genetic distribution worldwide. Similarly, all of the subjects recruited for the present study were found to be homozygous for the major allele producing a normal level of DNase 1L2 activity in all of the inactivating SNPs (S2 Table). Although the inactivating SNPs identified in the present study may not necessarily be distributed worldwide, the prevalence of psoriasis or other diseases resulting in parakeratosis is around 2–3%, indicating that the distribution of inactivating SNPs alone would not necessarily account for the prevalence of parakeratosis. Furthermore, other nucleases such as DNase II and TREX2 [5], as well as DNase 1L2, are thought to be responsible for DNA degradation in the epidermis. Recently, impaired autophagy has been postulated to contribute to the pathogenesis of psoriasis [33]. Therefore, it seems likely that each of the minor alleles for these SNPs may serve as one of genetic risk factors for parakeratotic skin diseases such as psoriasis and pityriasis rubra pilaris, characterized by aberrant retention of nuclear chromatin in cornified keratinocytes. In order to clarify any possible association of the inactivating SNPs with these skin diseases, the distribution of each SNP in the patient groups should be examined.

## Conclusion

We have demonstrated preliminarily that at least the SNPs causing loss of function of DNase 1L2, which constitutes the genetic background responsible for the pathogenesis of parakeratosis, can be estimated from the score obtained by the Polyphen-2 software program, which is a useful tool for predicting the possible impact of an amino acid substitution on the function of a human protein; a score of 1.000, corresponding to “probably damaging”, yields high predictive accuracy. In the present study, in order to effectively survey possibly pathogenetic SNPs in *DNASE1L2*, based on the damaging effects of the SNPs predicted using PolyPhen-2, all of the 28 SNPs thus assumed to be “probably damaging”, except for 10 SNPs examined previously [16], were selected from among the non-synonymous SNPs registered in the NCBI dbSNP and examined (S3 Table). Based on expression analysis of the DNase 1L2 bearing the amino acid substitutions corresponding to the SNPs, we identified 32 SNPs that abolished the activity, including 9 that had been characterized in previous studies [13–16]; the minor allele in each SNP was defined as a loss-of-function variant resulting in loss of DNase 1L2 function. Although these minor alleles were not distributed worldwide, thereby avoiding any marked reduction of the enzyme activity in human populations, it can be concluded that each of the minor alleles for these SNPs may serve as one of genetic risk factors for multiple skin diseases such as psoriasis, in which aberrant retention of nuclear chromatin occurs in cornified keratinocytes through incomplete DNA degradation. Similarly, it was clarified that all of the 19 SNPs originating from frameshift or nonsense mutations in *DNASE1L2* registered in the database result in loss of enzyme function, and are thus pathogenetic.

## Supporting information

S1 Table Primer sequence, annealing temperatures, and restriction enzymes for PCR-based genotyping for SNPs producing loss of funtion in *DNASE1L2*^a^. (XLSX)

S2 Table Genotype distribution and allele frequencies (95% CI) of non-synonymous SNPs producing loss of function in *DNASE1L2*. (XLSX)

S3 Table Summary on evaluation of all the non-synonymous SNPs in *DNASE1L2* predicted as a probably damaging (score = 1.000) SNP; genetic distribution, and effect of the corresponding amino acid substitution on the activity. (DOCX)
