# Supplementary material for: Survey of single-nucleotide polymorphisms in the gene encoding human deoxyribonuclease I-like 2 producing loss of function potentially implicated in the pathogenesis of parakeratosis
Source: PLoS One. 2017 Apr 10;12(4):e0175083. doi: 10.1371/journal.pone.0175083 (PMC5386265; doi:10.1371/journal.pone.0175083)
Supplement: S3 Table — (DOCX) [file pone.0175083.s003.docx]

**S3 Table** Summary on evaluation of all the non-synonymous SNPs in *DNASE1L2* predicted as a probably damaging (score=1.000) SNP; genetic distribution, and effect of the corresponding amino acid substitution on the activity

SNP Activity^a^ Genetic NCBI SNP database ^c^  ExAC database^d^

heterogeneity^b^ MAF Heterozygosity Allele Frequency

rs767698904 n.d. mono-allelic ‒ 0.000 2.17e^-05^

p.Arg23Pro; c.68G>C

rs753899553 0.23±0.044**** N.D. ‒ 0.000 2.28e^-05^

p.Ala26Thr; c.76G>A

rs369778646^e^ n.d. mono-allelic ‒ 0.000 2.36e^-05^

p.Asn28Asp; c.82A>G

rs779580244 0.50±0.19* N.D. ‒ 0.000 ‒

p.Gln59Leu; c.176A>T

rs200934792^e^ n.d. mono-allelic ‒ 0.000 0.001994

p.Arg94Trp; c.280C>T

rs745976143 n.d. mono-allelic ‒ 0.000 ‒

p.Phe103Cys; c.308T>G

rs773752842 n.d. mono-allelic ‒ 0.000 ‒

p.Phe125Val; c.377T>G

rs766981445 n.d. mono-allelic ‒ 0.000 1.91e^-05^

p.Pro129Leu; c.386C>T

rs202116861^e^ n.d. mono-allelic 0.003 0.0014 0.0003502

p.Lys133Asn; c.399G>T

rs370720965^e^ n.d. mono-allelic ‒ 0.000 0.0001776

p.Pro168Leu; c.503C>T

rs779344371 n.d. mon-allelic ‒ 0.000 4.59e^-05^

p.His170Tyr; c.508C>T

rs538832948 n.d. mon-allelic 0.0002 0.000 ‒

p.His170Gln; c.510C>G

rs760095531 0.63±0.053* N.D. ‒ 0.000 0.99e^-05^

p.Val177Met; c.529G>A

rs7614900229 n.d. mono-allelic ‒ 0.000 0.95e^-05^

p.Ala182Val; c.545C>T

rs200149634^e^ n.d. mono-allelic ‒ 0.000 0.000312

p.Leu183Pro; c.548T>C

rs750172458 n.d. mono-allelic ‒ 0.000 0.94e^-05^

p.Tyr184Cys; c.551A>G

rs766331933 0.68±0.14* N.D. ‒ 0.000 0.93e^-05^

p.Asp185Asn; c.553G>A

rs767403519 n.d. mono-allelic ‒ 0.000 3.56e^-05^

p.Leu202Pro; c.605T>C

rs775284591 n.d. mono-allelic ‒ 0.000 1.17e^-05^

p.Gly203Asp; c.608G>A

rs372443542^e^ n.d. mono-allelic ‒ 0.000 1.13 e^-05^

p.Asn206Lys; c.618C>G

rs753933779 n.d. mono-allelic ‒ 0.000 1.12 e^-05^

p.Ala207Val; c.620C>T

rs765446336 n.d. mono-allelic ‒ 0.000 1.06 e^-05^

p.Tyr211Cys; c.632A>G

rs200119394^e^ 0.10±0.054**** mono-allelic ‒ 0.000 0.0001674

p.Arg221His; c.662G>A

rs745600592 0.40±0.066*** N.D. ‒ 0.000 2.74 e^-05^

p.Arg223Thr; c.668G>C

rs776621988 n.d. mono-allelic ‒ 0.000 1.76 e^-05^

p.Trp230Leu; c.688G>T

rs761890431 n.d. mono-allelic ‒ 0.000 0.88 e^-05^

p.Trp230Cys; c.689G>C

rs201538561^e^ 0.15±0.033**** mono-allelic 0.0004 0.001 0.0002115

p.Leu231Phe; c.691C>T

rs751987582 n.d. mono-allelic ‒ 0.000 0.87 e^-05^

p.Ala236Pro; c.762G>C

rs753314287 n.d. mono-allelic ‒ 0.000 0.88 e^-05^

p.Val240Met; c.718G>A

rs745485267 1.3±0.36 N.D. ‒ 0.000 0.89 e^-05^

p.Asp244Asn; c.730G>A

rs771777101 n.d. mono-allelic ‒ 0.000 2.70 e^-05^

p.Cys245Tyr; c.735G>A

rs768519375 n.d. mono-allelic ‒ 0.000 1.84 e^-05^

p.Asp248His; c.742G>C

rs761783817 n.d. mono-allelic ‒ 0.000 0.96 e^-05^

p.Ile250Thr; c.749T>C

rs769743852 n.d. mono-allelic ‒ 0.000 1.00 e^-05^

p.Val251Ala; c.752T>C

rs376709672^e^ n.d. mono-allelic ‒ 0.000 7.87 e^-05^

p.Val268Met; c.802G>A

rs764602503 n.d. mono-allelic ‒ 0.000 1.39 e^-05^

p.Phe271Val; c.811T>G

rs562373958 0.18±0.031**** N.D. 0.0002 0.000 0.83e^-05^

p.Ala282Val; c.845C>T

rs113436938^e^ n.d. mono-allelic ‒ 0.000 ‒

p.Asp287Asn; c.859G>A

MAF, minor allele frequency.

^a^ The values are expressed as relative activity of each amino-acid substituted DNase 1L2 to that of the wild-type (1.00±0.20), representing the means ± standard deviation (*n*=4). P-value was calculated as differences between the activities of the substituted and wild type DNase 1L2 by means of the unpaired Student’s t-test; *,p<0.05, **p<0.01, ***p<0.005, ****p<0.001. n.d., the activity derived from the amino-acid substituted DNase 1L2 could be not detected under our assay conditions.

^b^ Genetic heterogeneity for each SNP was examined in our 16 different populations; N.D., not determined.

^c^ Taken from the NCBI SNP database (February, 2016); —, not presented in the database.

^d^ Taken from the ExAC database (February, 2017); —, not presented in the database.

^e^ Taken from our previous study [16].
